# P02.134. Effect of dispositional mindfulness on recovery from an acute laboratory stressor

**DOI:** 10.1186/1472-6882-12-S1-P190

**Published:** 2012-06-12

**Authors:** S Corey, P Moran, K Koslov, J Daubenmier, W Mendes, P Bacchetti, M Acree, M Kemeny, V Goldman, M Hall, E Epel, F Hecht

**Affiliations:** 1 Osher Center for Integrative Medicine, UCSF School of Medicine, San Francisco, USA; 2 Department of Psychiatry, UCSF School of Medicine, San Francisco, USA; 3 Department of Epidemiology and Biostatistics, UCSF School of Medicine, San Francisco, USA

## Purpose

The potential contribution of mindfulness to stress responses has not been fully described. The parasympathetic nervous system (PNS) can modulate stress responses and facilitate recovery from stressful events. Low levels of heart rate variability (HRV), a measure of the PNS, and suppressed vagal responses after stress tasks have been associated with increased morbidity, whereas vagal rebound has been related to better psychological adjustment and health outcomes. We hypothesized that dispositional mindfulness may facilitate recovery from a stressful laboratory task as indexed by greater increases in HRV once the stressor is complete.

## Methods

Pre-intervention measures of mindfulness (Five Facet Mindfulness Questionnaire; FFMQ), and acute stress (Trier Social Stress Test; TSST) were available for 5 of 6 waves of an obese adult population recruited for a diet and lifestyle study. From the TSST, the mean respiratory sinus arrhythmia (RSA), a key HRV measure, was calculated over 5 minutes at 3 time points: resting, acute stress (speech task), and recovery. HRV was calculated for the stress task (stress task-resting) and for recovery (recovery-stress task).

## Results

Of 154 participants from 5 of 6 waves of enrollment, 139 had complete data and were eligible for this sub-study. Mindfulness measures were not significantly associated with the average change in HRV between baseline and the stress task. In univariate analysis, higher scores for the Observing subscale of the FFMQ were associated with higher vagal rebound after the stress task (coefficient=0.32, 95% CI: 0.03, 0.62; p=0.029). Adjustment for age and gender resulted in a reduced coefficient=0.23 (CI: -0.06, 0.51; p=0.12).

## Conclusion

Mindfulness may contribute to recovery of the PNS from stressful events. Although adjustment for age and gender reduced the estimated association, it remained potentially clinically significant even though not statistically significant; further data may clarify this association and address whether changes in mindfulness with training enhance PNS recovery from stress.

